# A retrospective cohort study of pre-hospital agitation management by advanced paramedic practitioners in critical care

**DOI:** 10.29045/14784726.2022.12.7.3.8

**Published:** 2022-12-01

**Authors:** Nick Brown, Timothy Edwards, Ian McIntyre, Mark Faulkner

**Affiliations:** London Ambulance Service NHS Trust; London Ambulance Service NHS Trust; London Ambulance Service NHS Trust; London Ambulance Service NHS Trust

**Keywords:** acute behavioural disturbance, agitation, paramedic, pre-hospital, sedation

## Abstract

**Introduction:**

Pre-hospital clinicians can expect to encounter patients with agitation, including acute behavioural disturbance (ABD). These situations carry significant risk for patients and emergency medical services. Advanced paramedics within the London Ambulance Service (LAS) are frequently tasked to these incidents. At present, little evidence exists regarding clinical decision-making and management of this patient group. We sought to explore the demographics of patients presenting with potential ABD and quantify the degree of agitation, physical restraint, effectiveness of chemical sedation and any associated complications.

**Methods:**

A retrospective analysis of pre-hospital clinical records for patients coded with ABD and attended by LAS advanced paramedics between 1 October 2019 and 30 September 2020. Sedation assessment tool (SAT) scores were used as the primary outcome measure.

**Results:**

A total of 237 patient records were identified. Of the patients, 147 (62%) were physically restrained and 104 (44%) were chemically sedated. Sedation was more commonly administered where patients were exposed to physical restraint. High SAT scores were associated with the administration of sedative agents and at higher doses. Of patients undergoing sedation, 89 (85%) had a SAT score reduction of 2 points or a final score ≤ 0. The mean SAT score reduction was 2.72. Three cases of minor injury were reported following physical restraint.

**Conclusion:**

Advanced paramedics undertook sedation in less than half the cohort, suggesting that other strategies such as communication and positioning were utilised. Most patients were managed into a state between being restless and rousable, largely negating the need for ongoing physical restraint during hospital transfer. Appropriately trained advanced paramedics can utilise sedation safely and effectively in selected cases.

## Introduction

Pre-hospital care providers can expect to encounter patients with agitation. A spectrum of this cohort of patients will be seen, ranging from profound mania to those who respond well to simple verbal de-escalation techniques (Cole et al., 2018). Acute behavioural disturbance (ABD) is a term which can be used to describe a cohort of patients whose presentation is at the more extreme end of this spectrum. The UK Royal College of Emergency Medicine (RCEM) defines ABD (also known as ‘excited delirium’) as the presentation of features of acute delirium and hyper-adrenergic autonomic dysfunction associated with the following signs and symptoms (Gillings et al., 2016):

- extremely aggressive / violent behaviour;
- excessive strength / continued struggle despite restraint;
- insensitive to pain;
- acute psychosis with fear of impending doom;
- constant physical activity without fatigue;
- hot to touch / profusely sweating / inappropriate state of undress;
- hyperthermia;
- tachypnoea; and
- tachycardia.

Managing ABD in the pre-hospital setting is challenging. Pre-hospital clinicians can find themselves dealing with a fraught and chaotic environment. Safe patient assessment, treatment, handling and onward transfer can be significantly complicated by the ABD presentation, variable scene factors and difficult extrication. Additionally, clinicians will have to carefully oversee any restraint being undertaken by other agencies including police officers, security staff, relatives and members of the public. Some of these may have little or no training and experience in safe restraint.

The factors precipitating ABD can vary. However, themes have been identified including the use of illicit substances and alcohol and pre-existing mental health conditions (Gerdtz et al., 2020; Gonin et al., 2018; Vilke et al., 2012). Patients are often males around 30 years of age (Bond et al., 2019; Gonin et al., 2018; Vilke et al., 2012). The pathology is not well understood but is thought to involve surges in catecholamines as well as reduced reuptake of serotonin and dopamine leading to hyperthermia, metabolic acidosis, rhabdomyolysis and cardiac arrhythmias (Fraser et al., 2021). Factors associated with poor outcomes such as hypoxia, circulatory collapse and cardiac arrest include inappropriate or prolonged restraint, drug toxicity, underlying health problems and hot environments (ACEP Excited Delirium Task Force, 2009; Gill, 2014; National Institute for Health and Care Excellence [NICE], 2015). Mortality rates of 10% have been reported (Gillings et al., 2016).

RCEM recognises that prompt de-escalation is critical to reduce the risk of physiological collapse (Gillings et al., 2016). This includes managing environmental factors to achieve a calm scene and adopting best practice restraint techniques. If basic measures including reasoning with the patient are not effective, then pharmacological intervention may be indicated. Delivery of this intervention in the pre-hospital setting is often required to mitigate the significant risk of forcibly extricating an agitated and combative patient from a property or public place and conveying them to hospital in a moving ambulance, which may require ongoing restraint.

### Study setting

The London Ambulance Service (LAS) is the single emergency medical service (EMS) provider for the Greater London regions, serving a population of 8.2 million distributed throughout an area of 1579 km^2^. In excess of 2 million emergency calls are received and triaged using the Medical Priority Dispatch (MPDS™) system annually. The standard response to emergency calls consists of an ambulance staffed by emergency medical technician and paramedic staff with the addition of a paramedic rapid response vehicle for high-priority cases such as cardiac arrest.

In a smaller number of cases, the pre-hospital response is augmented by a solo advanced paramedic responder with additional postgraduate training and experience in critical care. At any one time, a maximum of five advanced paramedic resources are available across the London region and are selectively tasked to high-acuity cases such as cardiac arrest, major trauma and ABD.

Advanced paramedics are equipped with haloperidol, midazolam and ketamine administered under patient group directions (PGDs) to assist with the management of patients presenting with symptoms of extreme agitation and perceived to be unmanageable by non-pharmacological measures. NHS England (2021) describes PGDs as providing a ‘legal framework that allows certain registered healthcare professionals to supply and/or administer medicines to a defined patient group’. This approach is advocated in the College of Paramedics’ (2018) position statement and reflected in LAS advanced clinical operating procedures for sedation and management of ABD. During the study period, haloperidol up to 10 mg (intramuscular (IM)) or midazolam up to 10 mg (IM or intravenous (IV)) were the two pharmacological options available to advanced paramedics.

Determining the requirement for pharmacological intervention is in part based on the sedation assessment tool (SAT) (Table 1). The SAT score has been evaluated in the literature as a simple and rapid 7-point scale to measure the level of agitation/sedation in patients with ABD (Calver et al., 2011). Although decision-making is multi-factorial, patients scoring +2 or +3 might typically expect to be sedated.

**Table 1.** Sedation assessment tool.

SAT score	Responsiveness	Speech
**+3**	Combative/violent/‘out of control’	Continual loud outbursts
**+2**	Very anxious/agitated	Loud outbursts
**+1**	Anxious/restless	Talkative
**0**	Awake/calm/co-operative	Speaks normally
**-1**	Asleep but rouses to voice	Slurring / prominent slowing
**-2**	Responds to physical stimulation	Few recognisable words
**-3**	Non-responsive	Nil

SAT: sedation assessment tool.

### Aims

The objective of this study was to describe the epidemiology of pre-hospital ABD and its management by LAS advanced paramedics. Specific objectives were to:

1. describe ABD patient characteristics and settings;
2. define the locations in which restraint occurs and by whom;
3. identify causes attributable to the ABD presentation;
4. relate pharmacological interventions to SAT scores;
5. ascertain the effectiveness of sedation; and
6. highlight any complications.

## Methods

We undertook a retrospective evaluation of patient records coded as ABD and attended by advanced paramedics between 1 October 2019 and 30 September 2020. Data were collected from a bespoke advanced paramedic database completed manually in addition to the EMS patient record. This Microsoft Access™ database records details of additional interventions undertaken by advanced paramedics drawn from the EMS patient record, but contains no directly identifiable patient information. Pre-hospital diagnoses are recorded as part of each case record using pre-determined response options which include ABD.

Continuous data were analysed using descriptive statistics incorporating mean and standard deviation. Effective sedation was defined as a SAT score fall of two or a final SAT score of zero or less, as previously described in published literature (Calver et al., 2013). Approval for this service evaluation was granted by the LAS research and development approvals panel to ensure compliance with research and data legislation. The investigator (PO) was blinded to all patient identifiable data, negating the need for formal ethics approval. Neither patients nor the public were involved in the design or reporting of the study.

## Results

Our cohort comprised all 237 patients identified. Characteristics are detailed in Table 2. In total, 104 (44%) patients were administered sedation. Males comprised 73% (n = 174) of all patients; however, a similar proportion of both males (n = 74, 43%) and females (n = 30, 48%) were administered sedation. Mean age was similar between males and females. Most patients presented in a home environment. The location did not appear to be significantly associated with whether sedation was undertaken.

**Table 2.** Patient characteristics.

Demographics		Sedation administered	No sedation	Totals
	ALL	**104 (44%)**	**133 (56%)**	**237**
**Sex**	MALE	74 (43%)	100 (57%)	174
	FEMALE	30 (48%)	33 (52%)	63
**Mean age (SD)**		35.74 (15.28)	34.68 (14.32)	35.16 (15.67)
**Location**	HOME	64 (44%)	83 (56%)	147
	PUBLIC	31 (46%)	37 (54%)	68
	LEISURE	9 (41%)	13 (59%)	22
**Believed cause**	ALC/DRUGS	58 (50%)	58 (50%)	116
	MH	26 (38%)	43 (62%)	69
	NEURO	7 (47%)	8 (53%)	15
	INFECTION	13 (46%)	15 (54%)	28
	UNKNOWN	0 (0%)	9 (100%)	9
**Disposition**	ED	104 (49%)	119 (51%)	223
	OTHER	0 (0%)	14 (100%)	14

HOME refers to any residential address; LEISURE refers to social or entertainment location; PUBLIC refers to prison/police custody, and long-term and short-term healthcare facilities. ED: emergency department; MH: mental health; SD: standard deviation.

The presumed cause of almost half of ABD presentations was alcohol/drug related (n = 116, 49%). There was no significant variation in the proportions of patients undergoing sedation stratified by location. No patients where the underlying cause of ABD could not be determined underwent sedation. All patients who received sedation were transported to hospital. In total, 223 (90%) of all potential ABD patients were conveyed to an emergency department (ED). Of the 14 patients not conveyed to ED, 13 had a SAT score of zero on advanced paramedic assessment. One additional patient suffering a mental health crisis absconded from scene and was recovered by police and transported several hours later.

Table 3 highlights the relationship between physical restraint and patient location. Proportionally, restraint was undertaken most frequently in the home setting and less frequently in leisure settings. It was initiated in 147 (62%) cases and mostly by police or security staff (n = 121, 82%). Restraint by ambulance staff alone was provided infrequently. After intervention from an advanced paramedic, restraint was continued in 6/147 (4%) cases.

**Table 3.** Restraint location and by whom.

Restraint	Public/family	Police/security	Ambulance staff
**HOME**	3 (33%)	86 (71%)	7 (41%)
**PUBLIC**	4 (45%)	31 (26%)	6 (35%)
**LEISURE**	2 (22%)	4 (3%)	4 (24%)
**Total**	9	121	17

Sedation was administered in 97/147 (66%) cases where restraint occurred. This contrasts with seven (8%) episodes of sedation in the 90 unrestrained patients. The relationship between suspected cause of ABD and restraint varied across the groups. In the drug/alcohol category, application of restraint was proportionally higher, with 73% (n = 85) of patients restrained at some stage (Table 4). Patients suffering ABD from suspected neurological or infective causes were restrained less often.

**Table 4.** Relationship between restraint and suspected acute behavioural disturbance cause.

Restraint	Alcohol/drugs	Mental health	Neurological	Infection	Unknown
**YES**	85 (58%)	42 (29%)	5 (3%)	13 (9%)	2 (1%)
**NO**	31 (34%)	27 (30%)	10 (11%)	15 (17%)	7 (8%)

Higher SAT scores were associated with pharmacological intervention (Table 5). In 102/104 (98%) cases, sedation occurred when the initial SAT score was +2 or +3. On two occasions, sedation was undertaken when the SAT score was recorded as +1. Patients presenting with a SAT score of +3 were more likely to receive haloperidol and midazolam in combination and at higher doses. The maximum dose of 10 mg haloperidol was not exceeded in any patient. On three occasions, the maximum PGD dose for midazolam was exceeded after authorisation from an on-call physician. The first dose of midazolam was given via the IM route on 47 (52%) occasions and IV in the remaining 44 (48%).

**Table 5.** Relationship between sedation assessment tool score and pharmacological intervention.

DRUGS	No drugs	HAL only	MDZ only	HAL and MDZ	Totals	HAL (mean dose)	MDZ (mean dose)
**SAT +3**	2 (3%)	10 (18%)	9 (16%)	36 (63%)	57	6.75 mg SD = 2.55	6.13 mg SD = 2.74
**SAT +2**	7 (13%)	10 (18%)	15 (28%)	22 (41%)	54	6.67 mg SD = 2.55	5.45 mg SD = 1.54
**SAT +1**	26 (93%)	0	1 (3.5%)	1 (3.5%)	28	5 mg SD = 0	5 mg SD = 0

HAL: haloperidol; MDZ: midazolam; SAT: sedation assessment tool; SD: standard deviation.

All sedated patients had a pre- and post-sedation SAT score recorded. In 103/104 (99%) patients there was a reduction in SAT score post-sedation, with one patient remaining at SAT +3. Of the 102 patients who were sedated with an initial SAT score of either +3 or +2, 83 (81%) had a post-sedation score between +1 and -1. Thirteen patients had a post-sedation SAT score of -2 (12.5%), all of whom had had a pre-sedation score of +2 or +3. One patient had a post-sedation SAT score of -3 having had a pre-sedation SAT score of +3, although no respiratory or cardiovascular compromise was documented. Overall, 88 (85%) patients achieved either a SAT score reduction of two or a final score of zero or less post-sedation (Table 6).

**Table 6.** Sedation assessment tool score reduction post-sedation.

	Achieved SAT reduction of 2 or final score of 0 or less	Mean SAT score reduction (SD)
**Total**	89/104 (85.58%)	2.72 (1.29)
**Male**	65/74 (87.88%)	2.85 (1.34)
**Female**	24/30 (80%)	2.40 (1.11)
**Home**	55/64 (85.94%)	2.89 (1.36)
**Public**	26/31 (83.87%)	2.39 (1.14)
**Leisure**	8/9 (88.89%)	2.67 (1.22)
**Drugs/alcohol**	50/58 (86.21%)	3.02 (1.37)
**Mental health**	23/26 (88.46%)	2.23 (0.86)
**Neurological**	4/7 (57.14%)	2 (1.63)
**Infection**	12/13 (92.31%)	2.76 (1.09)

SAT: sedation assessment tool; SD: standard deviation.

There were no clinical complications recorded. Three cases of minor injury relating to restraint were reported. Two related to ambulance staff prior to advanced paramedic arrival and one to a patient with minor abrasions from police handcuffs.

## Discussion

We found that sedation was undertaken in less than half of cases where an advanced paramedic was dispatched to the scene for patients coded as ABD. Where pharmacological interventions were utilised, the majority of patients were sedated to achieve a conscious level between restless and rousable, negating the requirement for ongoing physical restraint until hospital arrival. The demographic of our cohort is consistent with those described in other studies (ACEP Excited Delirium Task Force, 2009; Gill, 2014; Vilke et al., 2012), specifically a higher numbers of males, a mean age in the mid-30s and a higher incidence of ABD related to drugs and alcohol.

Most of the cohort presented within a residential location over public or leisure settings. There are challenges in managing patients in all settings. Patients at home will generally be in more familiar and comfortable surroundings, perhaps with family available for support. However, egress from certain properties can be problematic, involving tight spaces, multiple flights of stairs or a small lift. Managing ABD in a public place or crowded leisure facility can involve greater unpredictability and volatility, although egress to an ambulance might be more straightforward. In reality, there are countless variations, all with multiple individual factors.

Less than half of patients suffering agitation underwent sedation. Although explicit reasons for non-sedation are not explored, it is likely that de-escalation was achieved without the requirement for pharmacological intervention in the majority of patients. Presumably, many patients could be reasoned with, and this may suggest that at the point an advanced paramedic arrived on scene they were not considered to meet the diagnostic threshold for ABD. Additionally, restraint may well have been adjusted after advanced paramedic arrival, removing painful stimuli and improving the patient’s behaviour and compliance.

Physical restraint is not without risk and needs to be managed carefully. NICE (2015) highlights that inappropriate restraint can lead to death and poor techniques also pose a risk to care providers. They advocate appropriate approaches to restraint in UK healthcare settings that incorporate patient positioning, ongoing monitoring, documentation and governance. Within the advanced paramedic system, all incidents of chemical and manual restraint mandate incident reporting, although compliance with the reporting mechanism was not examined.

Overwhelmingly, we found that the police were involved in restraining patients. Although police are trained in safe techniques, any restraint activity requires healthcare-professional oversight to advocate for the patient suffering ABD. These patients can be at the point of physiological collapse, so clinical monitoring and prompt identification of underlying pathology should be part of the system of safe patient management. Members of the public and some security staff may have no prior training or experience and this presents a greater risk to all parties. Any lay persons using force should therefore be replaced as soon as possible with trained personnel. However, it may be useful to recruit a family member as a familiar and reassuring voice to the patient, although this must be balanced against the risk of injury.

We found that restraint occurred most with suspected alcohol- and drug-related ABD cases. Application of physical restraint was associated with a greater likelihood of sedation by a factor of eight. Although the relationship between specific pharmacological interventions and the relinquishing of restraint is not explored in detail, in total only 6/147 (4%) patients continued to be restrained after advanced paramedic intervention, suggesting that sedation was largely effective in eliminating the requirement for ongoing restraint.

As expected, pharmacological intervention was associated with higher SAT scores. At the time of the study, advanced paramedics carried haloperidol and midazolam. Local guidelines suggest that a SAT score of +2/+3 may be a predictor of the need for sedation. Two patients fell outside this guidance, both with SAT scores of +1. Although sedation drugs are mandated under PGDs, advanced paramedics are empowered to apply clinical acumen with regards to patient presentation and which agents are appropriate. In all cases where dosing went beyond the confines of the PGD, input had been appropriately sought from an on-call physician.

Haloperidol is always given IM. However, there is an option of administering midazolam IM or IV. We found the IM and IV routes were used almost equally. IV cannulation in a patient who is very violent is difficult and can pose a significant risk. This observation suggests that those patients who received their first sedation dose IV may have been less animated. In a small case-control study by Calver et al. (2013) involving droperidol and midazolam, time to sedation was quicker in ABD patients exclusively given IM medicines over ABD patients predominantly administered IV sedation. In our study, 47 patients were given their first dose of midazolam IM. This figure is similar to the 45 patients who were given midazolam with a high SAT score of +3.

One view is that physical restraint can be applied until the patient arrives at hospital. This fails to take account of the danger presented to both patients and others on scene. Extrication of patients with ongoing physical restraint is challenging. The potential adverse clinical consequences of continued restraint in a patient who is close to maximum sympathetic stimulation require careful consideration. The end point for advanced paramedic sedation is compliance. The aim is not to render the patient comatose but to achieve sufficient conscious sedation to enable safe extrication which may include self-mobilisation in some cases. Despite the challenges of accurately titrating IM drug sedation, the data show most patients achieved a post-sedation SAT score between +1 and -1.

Despite several tools available for assessing ABD, there is no definite diagnostic test for the condition (Association of Ambulance Chief Executives & Joint Royal Colleges Ambulance Liaison Committee, 2019). In this study we used the SAT, and effective sedation was defined as a two-point reduction or a final SAT score of ≤ 0. This was achieved in 85% (n = 89) of sedated patients. This is marginally lower than the 92% (n = 157) reported by Calver et al. (2010), although this measure was applied to patients in a mental health unit administered droperidol, midazolam or haloperidol. Furthermore, in their study, 16 cases of complications involving oxygen desaturation and hypotension were reported.

We identified three complications associated with restraint. Patient and crew injuries have been reported in other studies (Calver et al., 2010). In our cohort, injuries were minor and consistent with flailing limbs or resistance to handcuffs. Dynamic assessment of patient handling and interaction is rarely more critical than when managing ABD to balance the opposing risks of allowing the patient full independence and overzealous handling. Indeed, the College of Paramedics (2018) in one of its seven recommendations around ABD management highlights the need for training and education around safe and legal restraint as well as recognition of the condition.

Over- and under-sedation is a risk due to not knowing exactly where the patient’s therapeutic margin lies. Over-sedation with midazolam can have significant respiratory and cardiovascular side effects (British National Formulary, 2021) which will need to be anticipated and built into the rescue plan. However, not gaining timely control of a restrained patient who is extremely violent and combative also carries appreciable risk. Within the advanced paramedic system, a subsequent change to the ketamine PGD now allows for ketamine sedation up to a maximum dose of 2 mg/kg IV or 10 mg/kg IM. Although ketamine does not have the same profile of side effects as benzodiazepines, potential complications include laryngospasm, hypersalivation, dysphoria and emergence phenomena. However, safe, rapid and effective control has previously been demonstrated in the pre-hospital environment (Cole et al., 2016). Future investigation should continue to explore the safety and efficacy of pre-hospital sedation undertaken by advanced paramedics, including ketamine.

## Limitations

Limitations are conspicuous by the retrospective nature of the study. We found association between variables that falls short of causation. Although records include patient status at hospital handover, no further follow-up was conducted. Additionally, time to adequate sedation is not recorded. The database used to retrieve records relies on advanced paramedics manually entering data post hoc. It is therefore possible that some cases have been omitted within the time period concerned, although database completion is monitored. The investigation was undertaken by those who work within the system, therefore reporting bias is conceivable.

## Conclusion

Advanced paramedics were selectively tasked to 237 patients with ABD over a one-year period with demographics similar to other literature describing this patient group. Both haloperidol and midazolam in isolation and combination were utilised to manage just under half of the patients within our cohort. However, advanced paramedics chose not to sedate patients in most cases, demonstrating that other non-pharmacological actions were likely effective. In all but six cases, restraint was discontinued after an advanced paramedic intervened, lowering the risk of complications in this vulnerable patient group. Despite the difficulties in titrating sedation to achieve an optimal response and not under- or over-sedating, most patients were managed into a state between being rousable and restless. Unsurprisingly, complications were identified but none that involved serious sequela in the pre-hospital phase. We found that appropriately trained advanced paramedics can selectively utilise sedation safely and effectively where required under an appropriate governance framework.

## Author contributions

NB: Researcher and main author. Final approval.

TE: Revision of manuscript and interpretation of data. Final approval.

IM: Acquisition of data. Final approval.

MF: Analysis and interpretation of data. Revision of manuscript. Final approval.

NB acts as the guarantor for this article.

## Conflict of interest

None declared.

## Ethics

Approval for the service evaluation was granted by the Clinical Audit and Research Unit of the London Ambulance Service NHS Trust and it was exempted from Ethics Committee approval. The researcher was blinded to all patient-identifiable data.

## Funding

None.
